# A comparison approach to explain risks related to X-ray imaging for scoliosis, 2012 SOSORT award winner

**DOI:** 10.1186/1748-7161-8-11

**Published:** 2013-07-02

**Authors:** Nicola Pace, Leonardo Ricci, Stefano Negrini

**Affiliations:** 1 BIOtech - Interdepartmental Center on Biomedical Technologies, Università di Trento, Trento, I-38100 Italy; 2 Dipartimento di Fisica, Università di Trento, Trento, I-38123 Italy; 3 Department of Clinical and Experimental Sciences, University of Brescia, Brescia, 25123 Italy; 4 IRCCS Don Gnocchi, Milan, 20100 Italy

## Abstract

**Background:**

X-ray imaging is frequently used as diagnostic approach for scoliosis in children and adolescents. X-ray procedures are considered as justified only when expected benefits exceed related risks. While benefits are well known to physicians, radiological risk awareness can be vague, impeding an optimal communication with patients’ parents and possibly leading to discomfort and anxiety. Objective of the study is the suggestion of a risk comparison approach for better communicating the radiological risks related to X-ray investigation of scoliosis.

**Methods:**

Starting point of the analysis is the Linear Non-Threshold (LNT) assumption for radiation stochastic effect, which states that for effective doses (E, Sievert – Sv) below 100 mSv, the probability of future stochastic damage is linearly related to E: absorbing two E’s in separate moments results in the addition of the risks related to each E. This allows to add E from different sources to calculate a cumulative risk of health detriment. Medline (Pubmed) was systematically searched in order to determine the average E delivered during X-ray investigation of scoliosis. Subsequently, the major natural sources of radiation were considered. The average yearly E due to natural sources was compared with E due to the imaging of the vertebral column.

**Results:**

E’s due to X-ray scoliosis examinations show a large variability: under 7 years of age, 0.03-0.54 mSv; 7–12 years, 0.11-0.80 mSv; 13–18 years, 0.17-1.09 mSv. Overall, 65% of the world population is expected to be exposed to an annual E between 1 and 3 mSv. More in detail, worldwide the total annual average E due to natural sources is 2.4 mSv (range 1–10), of which half originates from Radon exposure. Other sources are cosmic rays and ingestion and inhalation of radionuclides. For example, one flight between Europe and America accounts for 0.030-0.045 mSv because of exposure to cosmic rays.

**Conclusions:**

X-rays are carcinogenic and exposures to them always need to be justified and optimized in order to minimize the risks of health effects. However, the human body is continuously struck by radiations coming from natural sources. A useful element of comparison to evaluate E due to medical exposures in scoliosis can be then provided by the amount of E coming from natural sources. This comparison approach can play a role in the relationship between physicians and patients’ parents and lead to an improved awareness in patients’ parents.

## Background

Very often, physicians dealing with scoliotic children are asked by patients’ parents about the possibly dangerous effect of X-rays. The main goal of this paper is to suggest a comparative method to be used by physicians in order to help parents of children that are followed-up for spinal deformities to make an informed choice according to their own principles and risk evaluation.

Since their discover in 1895 by W. C. Röntgen, X-rays have been exploited for the inner exploration of human anatomy because of their non-invasive nature. Nowadays, X-ray imaging is one of the most used diagnostic techniques in modern medicine: worldwide, two-thirds of the 5 billion imaging investigations performed per year employ ionizing radiations [1]. In 2010, an estimated 182.9 million X-ray procedures were performed in U.S. hospital radiology departments [2]. In UK, in the period between 1998 and 2008 a 10% growth in the number of performed X-ray procedures has occurred, with an estimated 46 million X-ray procedures (including dental practice) in 2008 [3].

X-ray examination is the elective imaging procedure for the management of children with suspected scoliosis, in particular to confirm the presence of a potential spinal curvature, define the type of scoliosis, and evaluate the degree of curvature. Typically, for a single scoliosis survey, a posteroanterior (PA) or anteroposterior (AP), and a lateral radiography (LAT) of the spine are obtained. Occasionally, other projections are added, especially in patients undergoing first assessment or clinical treatment [4].

Despite of their widespread use, in some cases the appropriateness of the employment of X-rays is nowadays debated, and it raises concerns both from the clinicians’ and the patients’ side.

X-rays are high-frequency electromagnetic waves that belong to the group of the Ionizing Radiations (IR), which are able to ionize atoms and can cause damage to molecules. The effects of IR on biological systems have been investigated since the beginning of the 20^th^ century, when the first experiments with radioactive materials were carried out. The fact that IR exposure can lead to both early and late effects was soon recognized. Early effects refer to a large amount of conditions (e.g. burns, local necrosis, nausea and vomiting, cardiac pathologies, etc.), including death. Late effects mainly refer to the induction of cancer and hereditary effects. In 1945, the drop of the two atomic bombs on Hiroshima and Nagasaki (Japan) exposed a broad part of the population of those cities to a large amount of IR. The unmistakable and undeniable early and late effects of such exposures on individuals’ health led public opinion to the awareness of the risks related to IR. Subsequently, nuclear accidents such as the ones happened in Three Mile Island (USA, 1979), Chernobyl (former USSR, Ukraine, 1986) and Fukushima (Japan, 2011) enforced such awareness.

Mathematical models were introduced in radiological protection in order to predict the cancerogenic effects of the exposure of human body to IR [5-7]. Such models rely on data obtained by observations and follow-ups of exposed populations of Hiroshima and Nagasaki. These data are necessarily observational, retrospective, and non-randomized. Crucially, the amount of energy delivered by the IR released from the two atomic bombs to the observed population is much greater than the usual amount of energy delivered during X-ray examinations [8]; in addition, dosimetric models [7] assume that during the bomb explosions the whole body of subjects belonging to the target population was exposed: such total body irradiation is rather different from a typical medical exposure, which only targets determined organs. The combination of these two factors leads to a wide uncertainty about the actual magnitude of harmful effects due to X-ray exposures for medical purposes.

The wide amount of unfiltered information is a possible cause of the confusion shown by patients when called to express their perception of IR-related risks [9]. At the same time, as discussed in a recent study [10], a widespread, satisfactory knowledge of such risks is still to be achieved in the physician community making it difficult to manage the communication with patients with regards to the risk/benefit ratio of X-ray examinations.

From our own experience, when patients are children (potentially) affected by scoliosis or other spinal deformities, such sub-optimal communication is a frequent cause of uneasiness for patients’ parents, who can experience anxiety and apprehension in making a decision that is potentially harmful for their children.

Indeed, the human body is permanently struck by IR coming from outer space, the sun, the interior of the earth, and other natural sources. The amount of energy delivered by such sources is usually unknown by the general population. An approach oriented to compare medical with natural exposures could lead to a better understanding of the risks by patients’ parents, leading them towards a more aware decision making. Objective of the study is thus the comparison of IR-related risks due to X-ray procedures for scoliosis with IR-related risks due to natural sources.

## Materials and methods

### Unity of measure

In order to compare energies coming from different IR sources and striking different body parts, radiation protection professionals usually employ the concept of Effective Dose (E). E is a quantity introduced in 1990 by ICRP [5] to compare biological effects of different radiological procedures and to measure the potential harm that a certain amount of energy absorbed by the human body can produce. E can be calculated through computational methods (usually Montecarlo simulations) by taking into account the different radiosensitivity of different organs and the quantity of energy absorbed by each of them. The final value of E refers to the whole body, so that it is possible to compare risks related to different exposures to IR. E also keeps into account differences in biological effects produced by different kinds of IR (photons, charged particles, etc.). Its unit is the Sievert (Sv).

### Radiation dose, body response, and the Linear Non-Threshold model

While the way IR interact with inorganic matter is known and subsequent damages are deterministic, IR interaction with living matter is complicated by the fact that striking different cell structures leads to different early and late effects. Studies that were conducted by following up the atomic bomb survivors showed a linear dependence between E and cancerogenic IR effects; on the other hand, the extent of the dose–response relationship for non-cancerogenic late effects is still debated [11,12]. As stated above, the participants of these studies were exposed to average values of E much higher than those absorbed by patients exposed to X-rays. Thus, the linearity of the dose–response relationship for E under 200–500 mSv is still under investigation and remains undemonstrated. Among all the models developed for explaining the dose–response relationship for low radiation doses, the Linear Non-Threshold model (LNT) appears to be the most accepted and used for radiation protection regulation in many western world countries [6,13]. This model assumes that the linearity principle in dose–response relationship is valid also for E below 500 mSv, and that there is no theoretical inferior threshold under which radiation doses have no effects on human body. Under the LNT assumption, absorbing two separate E in separate moments results in a total risk that is the addition of the risks related to each E (additive property).

### Methods

Medline (Pubmed) was systematically searched in order to gather literature information about the average E delivered for scoliosis X-ray examinations. Inclusion criteria were: 1) articles reporting estimates of E due to PA and/or AP and/or LAT X-ray projections performed for the diagnosis/assessment of scoliosis; 2) articles in which E is estimated through Montecarlo simulations starting from primary clinical data; 3) articles published in English; 4) articles published in the period 1997–2012. Search terms were “effective dose” in conjugation with “scoliosis”. After the collection of the selected articles, data about average E for PA/AP and LAT projections, number of patients, and age of patients were extracted.

Subsequently, E due to natural sources were extracted by the reports of the United Nations Scientific Committee on the Effects of Atomic Radiations (UNSCEAR [14,15]). Estimates of average E both for a single activity (e.g. an intercontinental flight) and for a continuous exposure to a certain natural source (e.g. cosmic rays; annual amount of E is given) were considered.

On the basis of the results of the systematic review of literature, three reference levels of E were defined, to be used as reference points when different estimates of E for AP, PA and LAT projections are available. Finally, these three reference levels, as well as the minimum and the maximum estimated E for AP, PA and LAT projections, were translated into fractions of E due to different natural sources and presented for comparison purposes.

## Results

The systematic review of literature yielded 9 articles, of which 3 were included in this study because they met all the inclusion criteria [16-18]. Of these 3 included studies, 2 were focused on pediatric patients, and 1 considered both pediatric and adult patients (up to an age of 22 years). All the articles provided estimations for E due to AP, PA and LAT projections. For each article, E estimation was given according to patients’ age group.

For pediatric patients under 7 years, E ranged from 0.050 to 0.540 mSv for AP projections, from 0.030 to 0.252 mSv for PA projections, and from 0.120 to 0.421 mSv for LAT projections.

For pediatric patients between 7 and 12 years, E ranged from 0.270 to 0.800 mSv for AP projections, from 0.120 to 0.440 mSv for PA projections, and from 0.110 to 0.470 mSv for LAT projections.

For pediatric patients between 13 and 18 years, E ranged from 0.170 to 1.090 mSv for AP projections, from 0.290 to 0.490 mSv for PA projections, and from 0.260 to 0.540 mSv for LAT projections.

In general, E values show a large variability: for equal patients’ age, maximum and minimum estimated values of E can differ by more than 400%. AP projections showed estimates of E higher than PA projections.

Data extracted are reported in Table 1.

**Table 1:** Estimated E (Effective doses) for AP (antero-posterior), PA (postero-anterior) and LAT (lateral) projections for the investigation of scoliosis

**Author**	**Age of patients (y)**	**Subgroups**	**Number of patients**	**Mean AP projection E (mSv)**	**Mean PA projection E (mSv)**	**Mean LAT projection E (mSv)**
Mogaadi et al. [16]	0-15		86	0.503	0.252	0.421
	16-22		13	0.798	0.422	0.597
Gialousis et al. [17]	4-7	*Hospital A*	*	0.050	0.030	0.120
					
		*Hospital B*	*	0.220	0.210	N/R
					
	8-12	*Hospital A*	*	0.270	0.120	0.110
					
		*Hospital B*	*	0.440	0.240	0.410
					
	13-18	*Hospital A*	*	0.290	0.170	0.260
					
		*Hospital B*	*	0.470	0.250	0.290
					
Hansen et al. [18]	1-2		4	0.540	N/R	0.270
	3-6		9	0.540	0.230	0.270
	7-12		14	0.800	0.440	0.470
	13-17		22	1.090	0.490	0.540

*y* year of age. *In the paper by Gialousis et al patients were 31 in Hospital A and 105 in Hospital B, but the number of patients by age was not provided.

Data about natural sources of E were extracted from UNSCEAR reports (1993 and 2000) [14,15]. The worldwide annual average E for natural IR sources is estimated to be 2.4 mSv (range 1–10 mSv). This is composed by external IR sources (cosmic rays and terrestrial gamma rays, respectively 0.4 and 0.5 mSv) and internal IR sources (inhalation and ingestion of radionuclides, respectively 1.2 and 0.3 mSv). The huge range showed by the overall estimate of E is mainly due to Radon-222, a radioactive gas that contributes to the internal exposure due to inhalation (range 0.2 – 10 mSv).

Annual average E due to the exposure to cosmic rays at sea level is 0.270 mSv. This value is 2 times higher for inhabitants of Denver, USA (1600 m amsl), 3 times higher for inhabitants of Mexico City, Mexico (2200 m amsl) and 5 times higher for inhabitants of La Paz, Bolivia (3900 m amsl). Flying aboard an airplane at temperate latitudes accounts for an E from 5 to 8 μSv/h. Therefore, flying between Europe and America entails an E between 0.030 and 0.045 mSv.

So, because of cosmic rays, on average air travel crews are exposed to 3.0 mSv/yr, and, because of Radon-222, worldwide workers employed in above ground workplaces are on average exposed to 4.8 mSv/yr.

Data extracted from UNSCEAR reports [14,15] are reported in Table 2.

**Table 2:** Data extracted from UNSCEAR reports about annual average E (Effective doses) due to natural sources

**Activity**	**Annual average E (mSv/yr)**	**Radiation source**
Natural background due to external exposure (cosmic rays; terrestrial radionuclides)	0.9 (0.4; 0.5)	Cosmic rays, terrestrial radionuclides
Natural background due to internal exposure (inhalation; ingestion)	1.5 (1.2; 0.3)	Terrestrial radionuclides
Total natural background, worldwide	2.4	Cosmic rays, terrestrial radionuclides
Natural background due to cosmic rays, world average	0.380	Cosmic rays
Natural background due to cosmic rays, sea level	0.270	Cosmic rays
Natural background due to cosmic rays, 1600 m amsl	0.570	Cosmic rays
Natural background due to cosmic rays, 2200 m amsl	0.820	Cosmic rays
Natural background due to cosmic rays, 3900 m amsl	2.020	Cosmic rays
8 hours of flight (London-New York)	0.030 – 0.045 per flight	Cosmic rays

*E* Effective doses, *amsl* above mean sea level, *yr* year.

On the basis of the data collected, a table was developed in order to compare E due to radiological exposures for scoliosis and E due to natural sources (Table 3).

**Table 3:** Comparison between E (Effective doses) expected during radiological examinations typical of scoliosis diagnosis and treatment, and estimated E due to natural sources of Ionizing Radiations

**E (mSv)**	**Equivalent number of transatlantic flights**	**Percentage of annual natural background (%)**	Equivalent in time spent at 1600 m amsl (days)*	Equivalent in time spent at 3900 m amsl (days)*
**0.100**	2.7	4	64	18
**0.500**	13.3	21	320	90
**1.000**	26.7	42	641	181
**Min E for projections of Table 1 AP, PA, LAT**	1.3; 0.8; 3.2	2; 1; 5	32; 19; 77	9; 5; 22
**Max E for projections of Table 1 AP, PA, LAT**	29.1; 13.1; 15.9	45; 20; 25	698; 314; 383	197; 89; 108

*AP* antero-posterior, *PA* postero-anterior, *LAT* lateral, *Max* maximum, *Min* minimum, *amsl* above mean sea level.

* for people that live at sea level.

Reference E used in Table 3, namely 0.100, 0.500 and 1.000 mSv, were selected because they are representative of the estimated minimum, average and maximum E delivered for scoliosis X-ray examinations, respectively.

The minimum estimate of E due to AP, PA, and LAT projections corresponds to a value as low as 0.030 mSv (1 study, patients < 7 years, PA projection; see Table 1), whereas the average value amounts to about 0.4 mSv (standard deviation 0.2 mSv). This last value corresponds approximately to the same E that is absorbed by a reference person during a year because of the exposition to cosmic rays.

The maximum estimate of E due to AP, PA, and LAT projections corresponds to a value as high as 1.090 mSv (1 study, patients between 13 and 17 years, AP projection). Even if such value was far from the other available estimates, it was decided to keep the value of 1.000 mSv to be representative of high values of E related with X-ray procedures for scoliosis. For the case of 1.000 mSv, the proportions introduced above for the reference E of 0.100 mSv are to be multiplied by a factor 10.

## Discussion

Scoliosis in children is a complex disease that can be difficult to tackle, and diagnostic imaging plays a key role for the selection of the best therapeutic strategy to follow. Traditionally, physicians are proficient and effective in explaining benefits related to this diagnostic procedure. Nonetheless, besides the explanation of benefits, radiological procedures also entail the explanation of the expected related risks, and sometimes difficulties in having a satisfactory dialogue are highlighted by both physicians and patients’ parents.

Rather than discussing the radiological risk due to medical intervention by using epidemiologic considerations – an approach that is usually not well understood by laypersons –, we compared such risk with IR risks due to daily activities. The goal is to facilitate the achievement of a better awareness of risks due to IR and thus a better informed choice. The approach followed in this study might help physicians to improve their dialogue with parents’ patients, whose concern is that of exposing their children to procedures of unknown risks.

Every day, the human body is struck by IR coming from cosmogenic and terrestrial sources. This exposure accounts for a certain amount of E, that is possibly responsible of tissue damages. On first approximation, a similar kind of tissue damages are expected when the body is exposed to X-rays for medical purposes. Results show how the E due to X-ray exposures for scoliosis (in case of 1 AP – or PA – and 1 LAT projection) are comparable with those due to fractions of the annual exposure to natural sources. In particular, some estimates [14,17] indicate that E accumulated from the natural background in a period of 1–2 months accounts for the same E due to such radiological investigation. More in general, the estimates of E due to scoliosis radiological procedures for single exam tend to converge on values below 1 mSv, which is the same value of E delivered from the natural background in 5 months. It is also interesting to notice that E due to a standard scoliosis investigation is of the same order of magnitude as E due to a transoceanic return flight.

The fact that natural background is responsible of an annual E comparable with – and most of the time greater than – E due to radiological procedures for scoliosis may appear to be reassuring for both the physicians and the patients’ parents. It is important to specify, however, that the aim of such comparison is to provide an additional element to improve the communication between physicians and patients’ parents, rather than representing the only information about radiological risks to be delivered. In fact, two aspects have to be stressed: first, the approach exposed above relies on the validity of the LNT assumption (see Materials and Methods); second, under the LNT assumption, radiological exposures do represent a risk. Any exhaustive and effective explanation of these aspects cannot prescind from the justification and the optimization of X-ray employment. Indeed, radiological examinations should allow the collection of images with full diagnostic capabilities, with exposure levels *As Low As Reasonably Achievable* (ALARA principle [19]). This is particularly relevant in the case of periodic and frequent diagnostic investigations, which are common in the management of scoliosis.

Recently, a study showed an increase in breast cancer mortality among women which underwent frequent radiological examinations from 1925 to 1965 for spinal deformities [20]. However, as the authors of the publication highlighted, in recent years procedures, technologies and awareness of radiological risks have dramatically improved, with a consequent massive reduction of E delivered to patients. Moreover, caution must be used in comparing data about cancer mortality referred to decades prior to the implementation of breast cancer screening programmes with current data.

E that were estimated in the articles included in this work differ each other of up to 400%, even when patients of the same age group are considered. This effect can be associated to many factors. First of all, E due to a radiological examination is an estimated value instead of an actual measure. Models, parameters and calculation methods are characterized by uncertainties. Moreover, E depends on the acquisition parameters of the radiological device – for example, the higher the voltage and the current, the higher E –, on the radiological procedures, and on the characteristics of the diagnostic radiograph (newer devices usually include dose reduction technologies [17,18]). Finally, patients with different characteristics (i.e. age, sex, height and weight) are necessarily exposed to different amount of radiations in order to optimize the image.

Similarly, it must be noticed that the estimation of the worldwide average annual E due to natural sources is made by pooling populations and IR sources that show huge variability, mainly due to geographical and behavioral factors. Therefore, caution must be used in attributing a value of E due to natural IR sources to a single individual of the world population; in fact, E was calculated considering a Reference Person rather than an individual [6].

Data coming from literature cannot be representative of all the clinical situations in which radiological procedures are carried out. Because of this lack in generalization, E due to natural background were compared to the three reference doses of 0.1, 0.5 and 1 mSv, in addition to the estimated values of E due to medical procedures gathered in literature. Given a different specific value E attributed to a patient, it is possible to easily obtain the comparison with the average E due to natural sources by performing a simple proportion.

It is important that physicians who are called to prescribe radiological procedures for scoliosis collaborate with local radiation protection professionals in order to shed light on the E delivered to local patients. A full awareness of risks related to the prescribed radiological procedures must be considered as a key target for physicians [21].

## Conclusions

This paper was written in order to present a comparative method to evaluate risks related to X-ray examinations for scoliosis in children.

The human body is continuously struck by IR coming from natural sources, accounting for an E of 2.4 mSv per year (worldwide average, Reference Person). Such value shows how medical exposures due to scoliosis diagnosis and assessment amount to a part of the radiations that every year the human body absorbs. This information can enhance the proficiency of the communication between physicians and patients’ parents, driving these latter towards better informed choices.

It must be strongly highlighted that, even if the comparison approach proposed may lead to an improved awareness in patients’ parents, X-ray exposures always need to be justified and optimized, i.e. minimized, in order to minimize the risks for health.

## Competing interests

The authors declare that they have no competing interests.

## Authors’ contributions

SN proposed the clinical question. All the authors developed together the idea for the study. NP carried out the systematic search of Medline and drafted the manuscript. SN added clinically relevant information. LR provided technical revision. All authors read and approved the final manuscript.
